# The effects of segmentation algorithms on the measurement of ^18^F-FDG PET texture parameters in non-small cell lung cancer

**DOI:** 10.1186/s13550-017-0310-3

**Published:** 2017-07-26

**Authors:** Usman Bashir, Gurdip Azad, Muhammad Musib Siddique, Saana Dhillon, Nikheel Patel, Paul Bassett, David Landau, Vicky Goh, Gary Cook

**Affiliations:** 1 Cancer Imaging Department, Division of Imaging Sciences and Biomedical Engineering, King’s College London, London, SE1 7EH UK; 2 Stats Consultancy Ltd, 40 Longwood Lane, Amersham, Bucks HP7 9EN UK; 3 Department of Clinical Oncology, Guy’s and St Thomas’ NHS Foundation Trust, London, SE1 9RT UK; 4 Department of Radiology, Guy’s Hospital, 2nd Floor, Tower Wing, Great Maze Pond, London, SE1 9RT UK; 5 PET Imaging Centre and the Division of Imaging Sciences and Biomedical Engineering, King’s College London, London, SE1 7EH UK

**Keywords:** ^18^F-FDG PET, Segmentation, Inter-observer reproducibility, Non-small cell lung cancer, Prognosis

## Abstract

**Background:**

Measures of tumour heterogeneity derived from 18-fluoro-2-deoxyglucose positron emission tomography/computed tomography (^18^F-FDG PET/CT) scans are increasingly reported as potential biomarkers of non-small cell lung cancer (NSCLC) for classification and prognostication. Several segmentation algorithms have been used to delineate tumours, but their effects on the reproducibility and predictive and prognostic capability of derived parameters have not been evaluated. The purpose of our study was to retrospectively compare various segmentation algorithms in terms of inter-observer reproducibility and prognostic capability of texture parameters derived from non-small cell lung cancer (NSCLC) ^18^F-FDG PET/CT images.

Fifty three NSCLC patients (mean age 65.8 years; 31 males) underwent pre-chemoradiotherapy ^18^F-FDG PET/CT scans. Three readers segmented tumours using freehand (FH), 40% of maximum intensity threshold (40P), and fuzzy locally adaptive Bayesian (FLAB) algorithms. Intraclass correlation coefficient (ICC) was used to measure the inter-observer variability of the texture features derived by the three segmentation algorithms. Univariate cox regression was used on 12 commonly reported texture features to predict overall survival (OS) for each segmentation algorithm. Model quality was compared across segmentation algorithms using Akaike information criterion (AIC).

**Results:**

40P was the most reproducible algorithm (median ICC 0.9; interquartile range [IQR] 0.85–0.92) compared with FLAB (median ICC 0.83; IQR 0.77–0.86) and FH (median ICC 0.77; IQR 0.7–0.85). On univariate cox regression analysis, 40P found 2 out of 12 variables, i.e. first-order entropy and grey-level co-occurence matrix (GLCM) entropy, to be significantly associated with OS; FH and FLAB found 1, i.e., first-order entropy. For each tested variable, survival models for all three segmentation algorithms were of similar quality, exhibiting comparable AIC values with overlapping 95% CIs.

**Conclusions:**

Compared with both FLAB and FH, segmentation with 40P yields superior inter-observer reproducibility of texture features. Survival models generated by all three segmentation algorithms are of at least equivalent utility. Our findings suggest that a segmentation algorithm using a 40% of maximum threshold is acceptable for texture analysis of ^18^F-FDG PET in NSCLC.

## Background

Radiomics is the high-throughput extraction and analysis of computationally derived features from medical images [1]. Several studies have shown promising results in predicting tumour phenotype and prognosis with the help of radiomics, particularly those using features derived from texture analysis that describe intratumoural heterogeneity [2]. However, since it extracts information from the entire tumour, accurate tumour delineation by an appropriate segmentation algorithm is an important step in measuring tumour specific image parameters. A key property of a segmentation algorithm is that it should delineate tumour volume to a degree of accuracy sufficient to preserve the radiomic signature of the lesion. Using an inaccurate segmentation algorithm can cause incorrect tumour delineation by including adjacent non-tumour structures or exclude significant tumour regions. Both advanced and basic texture features alike are thus incorrectly estimated, and this incorrect estimation can potentially cause errors in prediction of tumour biology and patient outcome.

Besides accuracy, reproducibility is also an essential property for image segmentation algorithms and includes reproducibility across different acquisitions and reconstructions [3–6], multiple observers [7], different segmentation algorithms [8], and different bin ranges [6]. Lack of reproducibility, even in a few parameters, can impact the validity of serial measurements over time (e.g. to determine response assessment) and measurements performed on separate acquisitions (e.g. in multicentre trials).

Multiple segmentation algorithms exist, e.g. freehand algorithms (FH), thresholding-based algorithms (e.g. fixed thresholding at 40% of maximum intensity cut-off [40P]), and algorithms based on probabilistic classification of voxels into tumour or background (e.g. fuzzy locally adaptive Bayesian [FLAB]) [9]. In measuring tumour volumes, FLAB has been shown to be more accurate than threshold-based segmentation, especially for small or heterogeneous lesions (≤17 mm diameter) [8, 9]. The better results are probably because FLAB incorporates both the spatial context and intensity while classifying voxels, unlike threshold-based segmentation, which classifies voxels based purely on their intensities relative to the intensity of the most intense voxel in the region.

Nevertheless, a study on oesophageal cancer has shown no clear advantage in using either thresholding or FLAB in predicting patient survival—a frequent end-point of radiomics research [10]. Furthermore, there are no data directly comparing FLAB, FH, and thresholding in terms of inter-observer reproducibility of derived parameters. We hypothesised that different segmentation algorithms (FH, 40P and FLAB), despite potentially being discrepant in tumour volume delineation, are not significantly different in the prognostic power of derived texture features. If this hypothesis holds true, then the segmentation algorithm of choice would be the one that is most reproducible. The aim of our study was to test inter-observer variation of texture features in ^18^F-fluorodeoxyglucose (FDG) PET images across the three segmentation algorithms in a cohort of patients with non-small cell lung cancer (NSCLC) and to determine the effect of the different segmentation algorithms on the derived texture features’ prognostic performance.

## Methods

### Patients

Fifty-three consecutive patients (mean age 65.8 years; 31 males) with NSCLC treated with chemoradiotherapy (64 Gy with concurrent vincristine–cisplatin or vincristine–carboplatin chemotherapy) between 2007 and 2009 were included. All patients had single tumours. Most patients had locally advanced (stage III) NSCLC and were inoperable. Overall survival (OS) was recorded from the date of the ^18^F-FDG PET scan and was defined as the time in months between the PET scan and the date of death. Patients who were alive were censored at the time of the last clinical follow-up. A waiver of institutional review board approval was obtained for this retrospective analysis of anonymised data.

### Image acquisition and post-processing

^18^F-FDG PET/CT scans were performed at a median of 45 days (range 0–174 days) before treatment and all scans were acquired to the same protocol in the same institution on one of two scanners (Discovery VCT or DST, GE Healthcare, Chicago, USA) which are cross-calibrated to within 3% [11]. Patients fasted for at least 6 h before being injected with 350–400 MBq ^18^F-FDG intravenously. Ninety minutes (range 82–104 min) after tracer injection, PET images were acquired from the base of the skull to the upper thighs. Volumetric images were reconstructed using the ordered subset expectation maximisation algorithm with a slice thickness of 3.27 mm and pixel size of 4.7 mm. Low dose CT was acquired for attenuation correction at 120 kVp and 65 mAs without administration of oral or intravenous contrast agent. The reconstructed ^18^F-FDG PET datasets were imported into in-house texture analysis software implemented in MATLAB (Release 2013b, The MathWorks, Inc., Natick, Massachusetts, USA). Three readers, a radiation oncologist (GA), a radiologist (UB), and a nuclear medicine physician (GC), with 1, 8, and 25 years of  ^18^F-FDG PET imaging  experience, independently drew freehand regions of interest around the metabolically active primary lung tumours on each axial slice on the ^18^F-FDG PET scans to generate a volume-of-interest (VOI). Care was taken to exclude adjacent metabolically active structures, e.g. the heart and lymph nodes. Each FH VOI served as the template for automatic segmentation algorithms, i.e. 40P and FLAB. Before applying the respective algorithms, the FH VOIs were expanded by 5 pixels in three-dimensions to cover the entire tumour and some background non-tumour surrounding regions. The expanded VOIs were inspected again to ensure that no adjacent metabolically active non-tumour tissue had inadvertently been included as a result of expansion.

#### Forty percent of maximum threshold

The 40P VOIs were derived from the expanded VOIs by retaining only voxels showing activity equal to or greater than 40% of the maximum activity voxel inside the VOI [12].

#### Fuzzy locally adaptive Bayesian

Using the FLAB algorithm [9], the expanded VOI voxels were categorised into three classes representing tumour core, region of partial volume averaging around tumour core, and background. Voxels assigned to the background class were discarded, and the remaining volume, i.e. tumour core and region of partial volume averaging, was kept as the final FLAB VOI in accordance with a previous description of this algorithm [9].

### Texture parameters

The voxels of each VOI were resampled into 64 discrete bins of grey-scale values based on previous reports on optimum quantization schemes [6, 10]. The VOIs were then processed as three-dimensional matrices from which 83 texture parameters were derived: 6 model-based parameters (fractal) and 77 statistical parameters (20 first-order, 22 second-order and 35 higher-order).

### Statistical analysis

Statistical analysis was conducted using R 3.1 [13]. For inter-observer variability, all three readers’ datasets were used. The remaining analyses were performed on a single dataset of the most experienced observer (GC). Q-Q plots were examined to detect skewed distributions. Twenty - seven out of the 83 derived texture parameters showed highly positively skewed distributions; these parameters were log-transformed (base 10).

Before comparing derived texture parameters between segmentation algorithms, we compared measured tumour volumes by the different algorithms, since volume provides the foundation for all subsequent analyses. However, comparison of scalar volumes is inadequate because two techniques can give identical volumes yet have measured different regions and thus be discordant. Therefore, we used the Jaccard similarity index (JSI) to obtain a voxel-by-voxel comparison between VOIs drawn with different segmentation algorithms. JSI computes agreement between two VOIs drawn with different segmentation algorithms on a voxel-by-voxel basis. When both VOIs are identical, the JSI is equal to one, and when they are discordant, the JSI is equal to zero. The JSI was multiplied by 100 to obtain percent -agreement.

We used FLAB as the reference set, based on results from phantom studies [9], and FH and 40P were used to derive 2 sets of JSI for all 53 cases—1 set for FH/FLAB and 1 for 40P/FLAB. The JSI of FH/FLAB set was compared with the JSI of the 40P/FLAB set using the Mann-Whitney *U* test. The effect of tumour size on percent-agreement was assessed visually using scatterplots.

The ICC was used to measure the agreement between the three readers for each derived texture parameter. This yielded three sets of 83 ICC values—1 set per segmentation algorithm. To rate reproducibility of a segmentation algorithm, arbitrary cut-offs were used to denote high (ICC >0.85), moderate (0.7–0.85) and low (<0.7) reproducibility. To compare ICC values derived from different segmentation algorithms, 95% confidence intervals (CI) of pair-wise differences in ICC values were calculated from the same data resampled 100 times, using a bootstrapping approach [14]. A difference in ICC between two segmentation algorithms was considered non-significant if the CI included zero.

To determine if a given segmentation algorithm preserved sufficient relevant tumour information, we chose a subgroup of 12 texture parameters for univariate Cox regression analysis: metabolically active tumour volume (MATV), total lesion glycolysis (TLG), SUV_mean_, SUV_max_, SUV standard deviation, first-order entropy, grey-level co-occurrence matrix (GLCM) entropy, GLCM homogeneity, GLCM dissimilarity, grey-level size zone matrix (GLSZM) intensity variability, neighbourhood grey-tone difference matrix (NGTDM) coarseness and NGTDM contrast. We selected these variables after carefully reviewing relevant publications for their reported associations with patient survival [7, 10, 15–17]. The quality of each univariate cox regression model was assessed using two statistics: the Wald statistic was used to determine the statistical significance of the derived coefficient (cut-off *p* value: 0.05), whereas the Akaike information criterion (AIC) was used to determine goodness of fit of the model [18]. Our purpose was not to validate any of these texture parameters; we accepted them as valid biomarkers based on our literature review and merely used them to measure the performance of each segmentation algorithm. Thus, detailed survival analysis, i.e. analysis employing Kaplan-Meier curves and multivariate Cox regression, was not performed.

## Results

Patient demographic and clinical characteristics are listed in Table 1.

**Table 1:** Patient demographics and clinical characteristics

Patient characteristic	Value
Male:female	31:22
Tumour subtype	
Adenocarcinoma	21 (40%)
Squamous cell carcinoma	24 (45%)
Not specified	8 (15%)
T status	
T1	6 (11%)
T2	14 (27%)
T3	15 (28%)
T4	17 (32%)
Tx	1 (2%)
N status	
N0	11 (21%)
N1	4 (8%)
N2	33 (62%)
N3	5 (9%)
Tumour stage	
IB	3 (6%)
IIB	5 (9%)
IIIA	24 (45%)
IIIB	21 (40%)
Median interval between ^18^F-FDG PET and start of treatment (days)	45 (range 0–174)
Median radiotherapy dose (Gy)	64 (range 55–64)
Median chemotherapy cycles	4 (range 1–6)

### Effect of segmentation algorithm on tumour-volume estimation

**Fig. 1:**
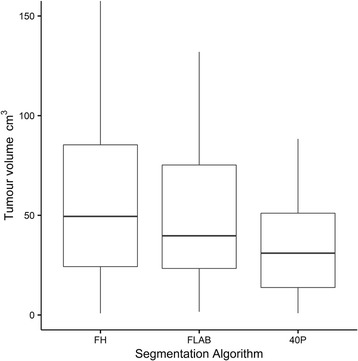
Boxplots comparing the three segmentation algorithms in volume measurement. The *boxes* represent the interquartile range (IQR). Horizontal lines through the boxes show median values. The *whiskers* represent values within 1.5*IQR. 40P = 40% of maximum intensity threshold. *FH* freehand, *FLAB* fuzzy locally adaptive Bayesian

Overall, 40P yielded the smallest volumes and FH the largest (Fig. 1).

**Fig. 2:**
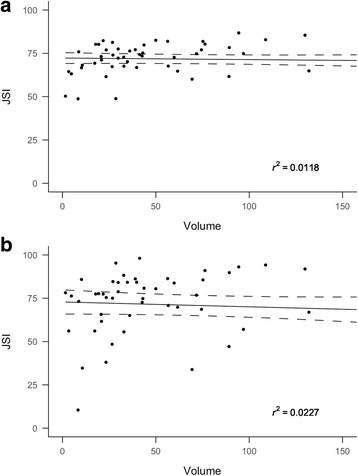
Lesion volumes computed with FLAB are plotted against JSI between FLAB and FH (**a**) and JSI between FLAB and 40P (**b**). *Slope lines* are shown along with 95% standard error of slope (*dashed lines*). *r* ^2^ values are displayed on the figures. The nearly *straight slope lines* and small *r* ^2^ imply that there is no particular trend to the degree of mismatch between FH and FLAB derived volumes over the range of tumour sizes. *JSI* Jaccard similarity index

Taking FLAB as the reference standard, mean JSI between FH and FLAB was 71.6% (range 48–87%; SD 9.4%) and between 40P and FLAB was 70.7% (range 10.5–98.1%; SD 21.1%). The difference between the two means was not statistically significant (*p* = 0.24). Furthermore, the percent-agreement of JSI did not appear to be related to tumour size (Fig. 2).

### Effect of segmentation algorithm on inter-observer reproducibility of derived texture parameters

Summary ICC statistics are as follows: FH (median ICC 0.77; IQR 0.7–0.85), FLAB (median ICC 0.83; IQR 0.77–0.86) and 40P (median ICC 0.9; IQR 0.85–0.92). Ranked on the basis of defined cut-offs, FH, FLAB and 40P showed high ICC (>0.85) in 20, 27 and 62 parameters, moderately high ICC (0.7–.85) in 40, 42 and 19, parameters and low ICC (<0.7) in 23, 13 and 2 parameters, respectively.

**Fig. 3:**
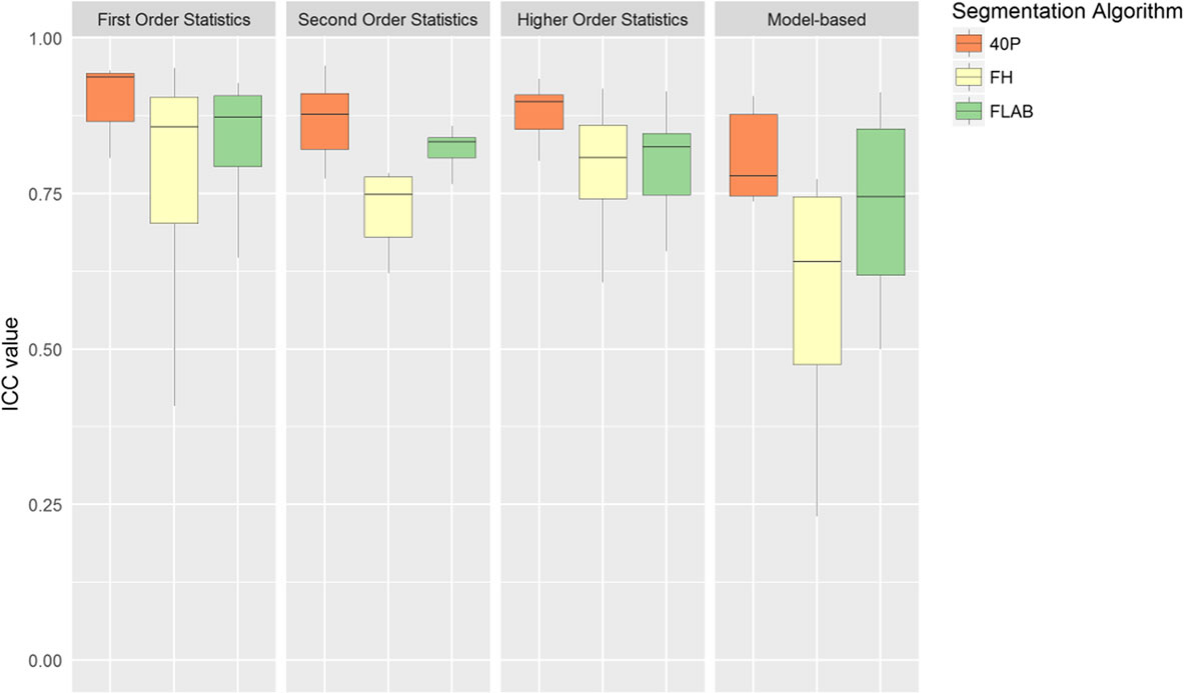
Boxplots comparing ICC values for the three segmentation algorithms over the four groups of texture parameters, i.e. first-order, second-order, and higher-order statistics, and model-based parameters. *40P* 40% of maximum intensity threshold. *FH* freehand, *FLAB* fuzzy locally adaptive Bayesian, *ICC* intraclass correlation coefficient

When ICC values of the segmentation algorithms were compared for individual texture parameters, the following observations were made: compared with FLAB, 40P showed greater ICC values for 77 of 83 parameters (statistically significant in 10 parameters); FLAB had higher ICC than 40P in the remaining 6 cases, 1 reaching statistical significance. Both FLAB and 40P had higher ICC than FH in 58 and 73 parameters, respectively, reaching statistical significance in 5 and 30 parameters, respectively. For SUV-range, FH had significantly greater ICC value than FLAB; compared with 40P, FH had significantly greater ICC in none. Group-wise comparison among texture parameters showed that the first-order histogram measures were the most reproducible for all segmentation algorithms (Fig. 3).

ICC values of commonly reported texture parameters are given in Table 2.

**Table 2:** Comparison of ICC values of 11 commonly reported texture parameters derived with 3 contending segmentation algorithms

Texture parameter	ICC FH (95% CI)	ICC FLAB (95% CI)	ICC 40P (95% CI)
TLG*	0.948(0.919–0.967)	0.939(0.906–0.962)	0.968(0.95–0.98)
SUV_mean_	0.9 (0.84–0.93)	0.91 (0.86–0.94)	0.94 (0.91–0.96)
SUV_max_	0.951 (0.925–0.97)	0.927 (0.887–0.954)	0.943 (0.911–0.964)
SUV Standard deviation	0.911 (0.865–0.945)	0.907 (0.859–0.942)	0.937 (0.903–0.961)
First-order entropy	0.745 (0.634–0.834)	0.775 (0.673–0.854)	0.87 (0.805–0.918)
GLCM entropy	0.767 (0.663–0.849)	0.779 (0.679–0.857)	0.868 (0.801–0.916)
GLCM homogeneity	0.782 (0.682–0.859)	0.833 (0.752–0.893)	0.912 (0.866–0.945)
GLCM dissimilarity	0.753 (0.644–0.839)	0.82 (0.734–0.885)	0.898 (0.845–0.936)
GLSZM intensity variability*	0.917 (0.874–0.949)	0.908 (0.86–0.943)	0.931 (0.894–0.957)
NGTDM coarseness	0.613 (0.469–0.738)	0.657 (0.522–0.77)	0.876 (0.814–0.922)
NGTDM contrast*	0.704 (0.581–0.805)	0.72 (0.601–0.816)	0.852 (0.779–0.906)

Note that ICC of MATV was not calculated as it was substituted for by JSI

*Variable was log-transformed

### Effect of segmentation algorithm on survival prognostication

Patients were followed up for a median 21.2 months (range 2.1–51.1 months). Median OS was 25.6 months. On univariate Cox regression analysis, 40P found 2 out of 12 variables, i.e. first-order entropy and GLCM entropy, to be significantly associated with OS; FH and FLAB found 1, i.e. first-order entropy (Table 3). For each tested variable, survival models for all three segmentation algorithms had comparable AIC values with overlapping 95% CIs showing equivalent fit to the data (Table 3).

**Table 3:** Results of univariate cox proportional hazards done on 12 commonly reported texture parameters

Texture parameter	FH		FLAB		40P	
	HR (95% CI)	AIC (95% CI)	HR (95% CI)	AIC (95% CI)	HR 40P (95% CI)	AIC (95% CI)
MATV^†^	1.1 (0.55–2.02)	220.81 (178.62–261.66)	1.06 (0.51–2.16)	220.8(176.18–259.29)	0.8 (0.39–1.61)	220.46 (178.15–259.25)
TLG^†^	1.0 (0.6–1.7)	220.84 (177.82–261.74)	1.0(0.57–1.73)	220.84 (173.1–257.46)	0.88 (0.53–1.47)	220.55 (176.2–264.39)
SUV_mean_	1 (1–1)	220.41(176.44–261.64)	1 (1–1)	220.24(178.26–261.34)	1 (1–1)	220.5(172.19–262.68)
SUV_max_	1 (1–1)	220.77 (174.54–259.51)	1 (1–1)	220.77 (178.24–258.84)	1 (1–1)	220.69 (175.34–261.62)
SUV standard deviation	1 (1–1)	220.23 (176.06–260.46)	1 (1–1)	220.33 (177.47–262.87)	1 (1–1)	220.26 (177.06–264.65)
first-order entropy	0.16 (0.04–0.74)*	216.12 (174.54–255.93)	0.2 (0.06–0.73)*	215.91 (175.69–259.35)	0.04 (0.005–0.328	212.47 (169.02–256.07)
GLCM entropy	0.56 (0.22–1.42)	219.53 (178.49–261.04)	0.45 (0.2–1.01)	217.8 (173.74–258.62)	0.21 (0.04–0.89)*	216.75 (173.21–256.01)
GLCM homogeneity	14.89 (0.12–1799.35)	219.68 (175.77–258.64)	32.51 (0.26–3999.05)	219.12 (173.93–256.75)	193.94 (0.02–2,314,503.14)	219.62 (176.21–259.9)
GLCM dissimilarity	0.836 (0.54–1.28)	220.18 (176.78–257.79)	0.75 (0.49–1.13)	219.09 (173.93–257.71)	0.83 (0.5–1.37)	220.32 (177.06–257.92)
GLSZM intensity variability^†^	0.46 (0.18–1.18)	218.24 (169.74–256.67)	0.65 (0.32–1.34)	219.55 (174.97–260.52)	0.72 (0.35–1.47)	220.07 (173.75–258.65)
NGTDM coarseness	0.88 (0.74–1.05)	218.64 (170.64–257.63)	0.9 (0.74–1.08)	219.6 (175.71–260.62)	0.82 (0.67–1.02)	217.62 (168.78–252.86)
NGTDM contrast^†^	0.24 (0.04–1.35)	218.45 (178.17–260.37)	0.18 (0.03–0.85)	216.85 (173.33–256)	0.46 (0.04–4.6)	220.42 (173.91–257.85)

*AIC* Akaike information criterion, *CI* confidence interval, *HR* hazard ratio

*p value <0.05

^†^Variable log-transformed

## Discussion

Our study found moderate to high reproducibility between observers, with 40P (median ICC 0.9) ranking highest, followed by FLAB (median ICC 0.83) and then FH (median ICC 0.77). Despite yielding smaller median volumes than FLAB and FH, and theoretically losing some texture information, 40P compared favourably with FLAB and FH by detecting a significant association between first-order entropy and survival. It found a second significant survival predictor (GLCM entropy) for which the other two segmentation algorithms were inconclusive.

For tumour volume measurements, we chose FLAB as proxy ground-truth in the absence of true measurements of resected specimens [10]. Although there is no consensus on segmentation algorithm suited for MATV delineation, a study has shown FLAB to be more accurate than a fixed threshold in tumour delineation over a range of phantoms (*n* = 6; sizes 10–37 mm) and simulated tumours (*n* = 3) [9]. There are no large studies comparing both techniques with resected specimen measurements. We found good voxel-by-voxel match (mean FLAB/FH and FLAB/40P match of 72 and 71%, respectively, *p* = 0.7)), uncorrelated with lesion size in our cohort of relatively large tumours. This means that in tumour sizes typically encountered, 40P- and FH-delineated volumes match FLAB-delineated volumes reasonably well and the degree of mismatch is stable over a range of tumour sizes. Segmentation with 40P generally estimated smaller tumour volumes. This is likely due to indiscriminate exclusion of voxel intensities below the fixed 40% threshold, such as those arising from low-activity tumour regions or tumour boundaries (subject to partial volume averaging with neighbouring tissue). FH, on the other hand, estimated larger tumour volumes. This is likely due to inclusion of some partial volume-averaged regions and some background region in physician - drawn contours.

In terms of inter-observer agreement, 40P ranked highest. We found FH to be inferior to other methods in inter-observer reproducibility, despite having a moderately good overall reproducibility (median ICC 0.86). The lower overall reproducibility of FH is due to its operator dependence, as opposed to the 40P and FLAB, which are semi-automatic. Other studies have also found FH to have moderate to good inter-observer variability [1, 19]. However, given that it is time-consuming and less reproducible than 40P and FLAB, we do not consider it the segmentation algorithm of choice.

We found a median ICC of 0.9 for 40P, which is similar to that reported in the literature [19]. 40P showed highest group-wise median ICC for all four groups of texture parameters, as illustrated in Fig. 3. Segmentation with 40P was especially robust in first-order statistical measures, for which it showed a median ICC of 0.94 compared with 0.86 for FH and 0.87 for FLAB. The reason for the higher reproducibility of 40P in first-order statistical measures is probably partly due to the inherent robustness of first-order statistical measures, as noted by others in CT [20] and PET studies [4, 7]. Furthermore, since 40P depends mainly on inclusion of the most active voxel in the region, and the calculation of the remaining volume is done automatically, it will not differ significantly between operators.

Comparing groups of texture parameters in terms of inter-observer reproducibility we found that all statistical measures were moderately to highly reproducible using any of the three segmentation algorithms, with first-order features ranking highest (median ICC for FH, FLAB and 40P: 0.86, 0.87 and 0.94, respectively). On the other hand, fractal dimension-related measures were least reproducible using FH (median ICC 0.64; IQR 0.47–0.74), FLAB (median ICC 0.74; IQR 0.73–0.85), or 40P (median ICC 0.77; IQR 0.75–0.88). While fractal dimension-related measures may have a role in lesion classification using CT [21], we did not find any reports supporting their role as prognostic biomarkers. Hence, these texture measures may not be useful in predicting prognosis.

Several texture analysis studies on patient survival have used freehand, threshold (40–50%) and FLAB algorithms [3, 7, 15, 22–24], highlighting different parameters in terms of their usefulness. However, these contending algorithms have not been compared in terms of effect on prognostic ability of derived texture parameters. In this regard, we found that 40P performed comparably to FH and FLAB in predicting overall survival when using first-order entropy. The fact that 40P discovered first-order entropy and an additional significant association between OS and a texture parameter (GLCM entropy), despite measuring generally smaller volumes, suggests that it preserves lesions’ radiomic signatures. Both first-order entropy and GLCM entropy have been shown in previous studies to have a potential role in predicting OS [7, 15, 16].

Our study has several potential limitations. First, the sample size is moderate but a larger sample may have revealed further prognostic associations. Nevertheless, as the main objective was to compare segmentation algorithms, the results remain informative. Second, we did not perform respiratory gating while performing ^18^F-FDG PET examinations of the lungs. Respiratory motion has been shown to add variability to measured texture parameters [25]. Third, we did not have histological ground-truth as a reference standard to compare the accuracy of volume delineation with the different algorithms. A few small studies using resected specimen measurements have found FLAB to be more accurate than fixed threshold [8]. Fourth, although our routine clinical protocol is to scan at 90 min post injection, many other departments scan at 60 min. It is possible that slightly different scan times may impact on segmentation volumes as there continues to be differential redistribution of FDG between benign and malignant tissue over time. Finally, we only tested the segmentation algorithms in an inter-observer reproducibility setting. Studies assessing test-retest reproducibility from different scanning sessions of various algorithms are necessary to validate best algorithms for multicentre trials and serial response-assessment examinations. It is possible that due to variation in maximum pixel intensity due to noise and reconstruction parameters in separate PET examinations, fixed thresholding may not be as reproducible as its contenders. While first-order statistical measures may not suffer significant differences in the test-retest setting [19], this potential shortcoming should be considered in radiomics research employing large numbers of higher-order variables.

## Conclusions

Compared to FH and FLAB, 40P is a robust segmentation algorithm for ^18^F-FDG PET texture analysis in NSCLC in terms of inter-observer variability, and it also produces the highest number of texture parameters associated with patient survival. It is therefore considered a clinically acceptable segmentation algorithm for texture analysis in NSCLC.

## Abbreviations

40P: Forty percent threshold-based segmentation
FH: Freehand segmentation
FLAB: Fuzzy locally adaptive Bayesian
GLCM: Grey-level co-occurrence matrix
GLSZM: Grey-level size zone matrix
ICC: Intraclass correlation coefficient
JSI: Jaccard similarity index
MATV: Metabolically active tumour volume
NGTDM: Neighbourhood grey-tone difference matrix
SUV: Standardised uptake value
TLG: Total lesion glycolysis
VOI: Volume of interest
